# Simplified and shortened French adaptation of a self-esteem contingency measure

**DOI:** 10.3389/fpsyg.2025.1393944

**Published:** 2025-05-30

**Authors:** Martin Robion, Sophie Berjot, Manon Balty, Céline Stinus, Kwamigan Ahondo, Charly Marie, David Bourguignon

**Affiliations:** ^1^Université de Reims Champagne-Ardenne, C2S, Reims, France; ^2^Université de Lorraine, PErSEUs, Metz, France

**Keywords:** self-esteem contingency, measurement, adaptation, validation, French

## Abstract

**Introduction:**

This study aims to validate a shortened and adapted French version of a self-esteem contingency measure specifically designed to evaluate how self-esteem depends on two fundamental psychological needs: competence and affiliation. To ensure a clear understanding and broaden the tool's validity across diverse populations, it was tested among three groups: students, job seekers, and employees.

**Methods:**

Four samples participated in the survey: students (*N* = 221, *N* = 507), job seekers (*N* = 270), and employees (*N* = 328). Participants completed the adapted self-esteem contingency scale along with other selected scales to assess convergent and discriminant validity. Exploratory factor analysis (EFA) and confirmatory analyses (CFA, ESEM, Bifactor-CFA, and Bifactor-ESEM) were conducted to explore the scale's structure.

**Results:**

The EFA revealed a two-dimensional structure, while the confirmatory analyses suggested a bifactorial model composed of one global factor and three specific factors: contingency regarding competence, self-criticism, and contingency regarding relationships. The bifactorial model demonstrated good internal consistency across all groups and satisfactory temporal stability. Correlation analyses with other constructs supported the convergent and discriminant validity of the scale.

**Conclusion:**

Overall, the shortened and adapted French version of the self-esteem contingency measure is a valid and reliable instrument. It assesses global self-esteem contingency while accounting for the specificities related to the needs for competence and affiliation. This dual focus enhances the scale's applicability in both research and intervention contexts.

## 1 Introduction

While it is widely acknowledged in the literature that possessing high self-esteem is associated with various positive outcomes in terms of psychological adaptation and mental health, in contrast to low self-esteem (Crocker and Park, 2004; Stinson et al., 2008; Orth and Robins, 2022), a substantial body of research has nuanced these findings. Indeed, beyond the level of self-esteem, it is the secure vs. fragile/insecure nature of self-esteem that plays a key role in its impact on the individual (Kernis et al., 1989; Kernis, 1993; Kernis et al., 1991; Jordan and Zeigler-Hill, 2013). Secure self-esteem refers to a deep sense of personal value based on realistic self-perceptions, as well as an authentic and stable self-expression that is not easily undermined (Rogers, 1959). Individuals with secure self-esteem are aware of their weaknesses but don't fundamentally question their overall sense of personal value when faced with failure or rejection. They don't need to feel superior to others, elevate their self-esteem, or seek external validation. Conversely, fragile or insecure self-esteem is defined by a sense of personal worth based on unrealistic self-perceptions and contingent on compliance with meeting certain standards or criteria for what defines a valuable person. The self-esteem of insecure individuals thus needs continuous validation from others to sustain a sufficiently high level (Deci and Ryan, 1995; Kernis, 2003).

The contingency of self-esteem, which is of interest in this context, is one of the indicators of fragile/insecure self-esteem (Jordan and Zeigler-Hill, 2013). It refers to the dependence of individual self-evaluations on standards of performance, approval, or acceptance by others. Thus, individuals whose self-esteem is contingent only value themselves (i.e., have positive self-esteem) when they meet these standards, namely when they succeed and/or are accepted by others. They are therefore frequently anxious and preoccupied with these goals, are particularly reactive to situations preventing their achievement, such as failures or rejection, and engage in various strategies to protect their self-esteem (Kernis, 2003). They tend to avoid failures, adopt prevention or performance goals, disengage from domains in which they don't succeed, exhibit lower autonomy, and attribute failure to external factors. Their interpersonal relationships are generally of lower quality (see Crocker and Park, 2004 for a review).

In line with these results, several authors have explored this promising concept, proposing various definitions and operationalizations. However, most of the scales used to assess this concept are not translated in French or are lengthy and relatively complex to understand. The objective of the present studies is, therefore, to propose a brief and simple scale assessing the contingency of self-esteem, potentially applicable to diverse populations in different contexts. Thus, our choice has been directed toward a scale evaluating the two primary sources of standards that contingent individuals may target, namely performance and acceptance by others.

For Deci and Ryan (1995) “Contingent self-esteem refers to feelings about oneself that result from—indeed, are dependent on matching some standard of excellence or living up to some interpersonal or intrapsychic expectations.” According to these authors, the contingent self-esteem develops in response to past experiences in which the individual could not act autonomously, competently, and could not feel accepted (Deci and Ryan, 1995). In other words, during past experiences that could not satisfy or frustrate individuals' fundamental psychological needs. Experiencing such a context is likely to lead individuals to regulate their behavior by internalizing these approval standards in the pursuit of their goals (i.e., through introjection). This internalization can make their self-esteem contingent on achieving these standards, which becomes a necessary condition for maintaining positive self-esteem.

In line with this definition but focusing more on the dependent nature of self-esteem, Paradise and Kernis (1999) developed a 15-item scale assessing the degree to which individuals' self-esteem “depends on its correspondence with certain standards, achievements, and evaluations from others” (Kernis and Goldman, 2006). In other words, the scale assesses how much an individual's self-esteem is tied to their perceived success in various dimensions, including attractiveness and intelligence. Its psychometric qualities appear satisfactory, as does its predictive validity. Indeed, contingency measured using this scale seems to be significantly and negatively associated with individuals' psychological health and wellbeing (Kernis, 2003; Kernis and Lakey, 2010). However, this scale does not distinguish the domains on which the individual bases his or her evaluation, not does it specify the standards used to develop it (personal standards or societal standards), and the origin of this source of information (achievements or approval of others).

In line with the definition by Deci and Ryan (1995), Crocker and Wolfe (2001) adopted a contingency approach that focuses on the domains upon which individuals base their self-esteem. In this perspective, the value individuals attribute to the self depends on their perception of failure or success in the different domains, considered important for their self-definition. To determine this contingency, Crocker et al. (2003) proposed a 35-item scale assessing 7 domains of contingency among students distinguishing two types of contingencies: contingencies related to external domains of self-worth (e.g., physical appearance, competition, academic success) that depend on the approval of others, and contingencies related to internal domains of self-worth (e.g., religion, virtues). This scale presents good psychometric qualities, particularly in terms of convergent, discriminant, and predictive validity. Thus, the authors suggest that the predominance of external contingencies over internal ones is likely to weaken self-esteem. For instance, Sargent et al. (2006) have shown that external contingencies among students are predictive factors for an increase in depressive symptoms during the academic year, which is not the case of internal contingencies. The latter are more strongly associated with prosocial behaviors, which are conducive to the creation of supportive relationships and, in this sense, protective against potential adverse effects of contingency (Crocker and Park, 2012).

If these two scales are commonly used to measure the overall level (Paradise and Kernis, 1999) or specific domains (Crocker et al., 2003) of self-esteem contingency, two other scales have subsequently been proposed to address some limitations, particularly those associated with the approach of Crocker et al. (2003). Williams, Schimel, Hayes, and Martens (Williams et al., 2010) for example, argued that while it is indeed important to distinguish contingencies according to external and internal domains, their scale is unable to identify whether the importance attached to these domains is driven by extrinsic reasons (e.g., success or approval from others) or intrinsic reasons (e.g., interest in the domain). According to Williams et al. (2010), only domains driven by extrinsic reasons can make self-esteem contingent. Thus, self-esteem is truly contingent only when it depends on socially imposed standards, from which individuals seek approval and/or success.

To challenge this limitation, the authors proposed a unidimensional scale of 20 items (translated into French by Leboeuf and Losier, 2012) specifically measuring individuals' dispositional tendency to be focused on extrinsic contingencies, more precisely to “evaluate oneself based on extrinsic criteria over which the individual has little control” (Leboeuf and Losier, 2012). Although it does not distinguish specific domains, this new scale focuses on a general dependence based on extrinsic sources of self-esteem, regardless of the domain and source (achievements vs. approval from others). This is currently the only tool validated in the French language.

More recently, Wouters et al. (2016) developed another measure of self-esteem contingency specific to domains and intended for students. An important contribution of this scale compared to the scale of Crocker et al. (2003), is the fact that this scale considers the valence of events associated with this contingency. According to Wouters et al. (2016), self-esteem contingency is not necessarily detrimental and can even be beneficial when it reflects an enhancement of self-esteem in response to successes in important domains. In such cases, self-esteem contingency is positively associated with overall self-esteem. Where contingency becomes particularly harmful, however, is when it relates to negative events such as failures or rejection from others.

Finally, Johnson and Blom (2007) contest the postulate of Crocker et al. (2003) that all individuals possess self-esteem contingent on one or more domains. This disagreement originates from the distinction proposed by Johnson and Forsman (1995) between basic self-esteem and earning self-esteem. While the former is acquired during early interactions and roughly corresponds to the global trait self-esteem described by Rosenberg (1965) (see Dåderman and Basinska, 2013), earning self-esteem develops later when individuals have not been able to acquire a sufficient level of basic self-esteem. It is in response to this insecurity that the contingency of their self-esteem takes shape, leading them to seek reassurance through two sources of validation: the need for competence and the need for acceptance in relationships. This approach does not exclude the possibility that individuals with high basic self-esteem may still seek enhancement through these sources of validation. However, these individuals are generally less dependent on them because they benefit from a more secure attachment.

Finally, following this approach, Johnson and Blom (2007) developed two scales to measure the degree of self-esteem contingency on two of the three fundamental needs described by Deci and Ryan (1985, 1991): the need for competence (i.e., CBSE, Competence based self-esteem) and the need for acceptance in social relationships (i.e., RBSE, Relation based self-esteem).

The Competence Based Self-Esteem (CBSE) scale measures attitudes related to the belief that personal worth is defined by achievements and failures, as well as the attainment of perfection criteria (Deci and Ryan, 1995; Johnson and Blom, 2007). It consists of two sub-dimensions, one related to the dependence of self-esteem on achievements/performance (e.g., “I feel worthwhile only when I have performed well”), and the other related to self-criticism in case of failure and the feeling of dissatisfaction associated with the pursuit of these achievements (e.g., “When I have failed in an exam or in another context performed worse than I expected it has made me doubt my self-worth”). By forming indices, the authors identified a third sub-dimension related to comparisons with others in terms of success (“e.g. Other people's success makes me push myself even harder”).

The Relation Based Self-Esteem (RBSE) scale, on the other hand, measures attitudes related to the perception that personal worth depends on the acceptance and rejection from others. This dependency manifests as a vulnerability to rejection (e.g., “I am sensitive to signs of dislike and rejection from others”), a constant need for support and love from others (e.g., “It is important for my self-esteem to be loved”), and a tendency to be compliant, even if this implies to frustrate one's own needs and emotions to avoid rejection (e.g., “I tend to suppress my own needs and emotions to make others feel good”) (Johnson and Blom, 2007). Each of these three elements is represented by a sub-dimension of the RBSE.

These two scales by Johnson and Blom (2007) exhibit good psychometric qualities. The study conducted by these authors demonstrates that the model presenting contingency in two factors (i.e., RBSE and CBSE) shows good fit indices (χ*^2^8* = 11.16, *p* = 0.19; RMSEA = 0.04; NFI = 0.98; NNFI = 0.99; CFI = 0.99; GFI = 0.98). Additionally, both the CBSE and RBSE each have good internal consistency (α = 0.89; α = 0.88) and good temporal stability after a 5-week test-retest interval (*r* = 0.93; *r* = 0.80). Finally, these two scales exhibit good convergent and discriminant validity. For instance, their study shows that the CBSE significantly and positively correlates (*r* = 0.41, *p* < 0.01) with the pursuit of “toxic” achievement (Birks and Roger, 2000), which is not the case for the RBSE (*r* = 0.01). In contrast, only the RBSE significantly and positively correlates (*r* = 0.36, *p* < 0.01) with affiliation need (Hill, 1987).

These links are consistent with those observed in other studies using these two scale or dimensions, indicating that the active pursuit of success and performance by individuals strongly contingent on their competence is associated with a critical and unforgiving attitude toward their weaknesses. These negative attitudes lead them to exert even more effort to maintain their self-esteem, exposing them to increased risks of stress and burnout (Blom, 2012). On the other hand, individuals who depend on emotional support from others tend to prioritize the needs and emotions of others over their own to avoid rejection. This dependence on the approval of others is reflected in a passive and less adaptive behavioral style, causing individuals to experience anxiety and relational tensions that they struggle to regulate (Johnson, 2011; Gillath et al., 2005).

In conclusion, while authors generally agree to define contingency as a dependence of self-esteem on the achievement of reference standards and interpersonal or intrapsychic expectations (Deci and Ryan, 1995), there still seem to be some disagreements regarding its sources, domains of application, and operationalization. While some authors emphasize the link between self-esteem and individual outcomes such as failures/successes or acceptance/rejection (Paradise and Kernis, 1999), others focus more on the source of self-esteem, whether it is the domains considered important (Crocker et al., 2003) or the fact that this source of self-esteem is extrinsic (Williams et al., 2010).

The tool developed by Johnson and Blom (2007), on the other hand, is also consistent with the definition by Deci and Ryan (1995), maintaining the dependence link between self-esteem and standards, which are external and extrinsic. However, it explicitly distinguishes (unlike Kernis' scale) two sources related to identity needs: the needs for competence and affiliation, transcending domains and associated with distinct consequences (Johnson and Blom, 2007; Deci and Ryan, 2000). Finally, this operationalization of self-esteem contingency is also consistent with the self-determination theory (Ryan and Deci, 2017), providing an opportunity to better understand not only its causes but also its consequences on the wellbeing and motivation of individuals in general and specific populations (e.g., employees, job seekers). According to us, this scale represents a good compromise among the available instruments. However, it should be noted that while the authors emphasize the distinction between the two sources of contingency (i.e., competence, affiliation) by creating two separate scales, these sources positively correlate with each other and can be considered as two dimensions of self-esteem contingency.

The aim of these studies is to translate, adapt and validate the two contingent self-esteem scales developed by Johnson and Blom (2007). This adaptation aims to simplify and shorten this tool in a way that makes it understandable to all types of populations (e.g., adolescents, pre-adolescents, individuals with limited language proficiency). However, like most contingency scales and due to the complexity of the construct, the items are often challenging for everyone to comprehend. Therefore, we prioritized simplicity in the translation of the items.

The two scales developed by Johnson and Blom (2007) present certain limitations in their operationalization regarding our research objectives. Specifically, these scales evaluate self-esteem contingency not only through its dependence on affiliation and competence but also by integrating attitudes and behaviors resulting from this dependence. Incorporating behavioral reactions into the measurement of contingency itself creates a conceptual overlap that undermines the clarity of the construct.

To address this issue, we decided to exclude two sub-dimensions that, in our view, reflect coping strategies rather than core aspects of self-esteem contingency. These are the “comparison to others” sub-dimension of Competence Based Self-Esteem (e.g., “Other people's success makes me push myself even harder”) and the “compliance” sub-dimension of Relation Based Self-Esteem (e.g., “I tend to suppress my own needs and emotions to make others feel good”).

Beyond the conceptual overlap, the “comparison to others” sub-dimension presents additional methodological challenges. Its three items do not form a coherent factor in confirmatory factor analyses and were primarily grouped to balance the scale's dimensions. This grouping is problematic, as only two items (“Other people's success makes me push myself even harder” and “Other people's success is threatening”) explicitly involve social comparison, while the third (“I easily get restless if I have nothing at hand to accomplish”) lacks a direct reference to others. Furthermore, the latter item poses translation difficulties (e.g., “I easily get restless if I have nothing at hand to accomplish”) that could compromise the scale's content validity. Additionally, these items exhibit the lowest factor loadings within the Competence Based Self-Esteem sub-dimension (0.44 < *r* < 0.50), further supporting their removal.

Regarding the “compliance” sub-dimension, we believe that limiting individuals' reactions to social approval to this single strategy is too restrictive. In fact, individuals with contingent self-esteem may respond to threatening information with reactions opposite to compliance, such as blaming others for their failures, derogating those who criticize them, or distorting or denying unfavorable information (Deci and Ryan, 1995; Kernis, 2003). Moreover, recent findings by Enjaian et al. (2017) suggest that self-esteem contingency based on social approval is not inherently linked to conformity, further supporting our decision to exclude this sub-dimension.

After excluding these items, our simplified scale consists of 14 items: 9 for Competence Based Self-Esteem and 5 for Relation Based Self-Esteem. This streamlined version was subsequently validated across diverse populations.

## 2 Material and methods

### 2.1 General procedure of the studies

#### 2.1.1 Factorial structure and psychometric qualities

Two studies were conducted to examine both the factor structure and the psychometric properties of the scale. The first study involved an exploratory analysis with a student sample (*N* = 221) and assessed the scale's concurrent validity. The second study compared various models, including Confirmatory Factor Analysis (CFA), Exploratory Structural Equation Modeling (ESEM), bifactor-CFA, and bifactor-ESEM, across three additional samples: students (*N* = 507), unemployed individuals (*N* = 270), and employees (*N* = 328). For each sample, we compared the fit of different self-esteem contingency operationalizations, considering both unidimensional (i.e., global) and bidimensional forms (i.e., Competence Based and Relation Based Self-Esteem) while testing for the presence of a general factor. Finally, we assessed the scale's psychometric properties, focusing on convergent, discriminant, and predictive validity, as well as temporal stability across samples.

Regarding concurrent validity, we hypothesized that the measures of self-esteem contingency (i.e., global, competence-related, relationship-related) would be strongly and positively correlated with Leboeuf and Losier's (2012) contingency scale. However, we expected these correlations not to exceed 0.70, given that these two scales propose a different operationalization of self-esteem contingency: a focus on the need for competence and acceptance in the case of our short version scale inspired by Johnson and Blom (2007) and, a focus on the achievement of extrinsic criteria in the case of Leboeuf and Losier's (2012) scale.

Regarding convergent validity, we hypothesized that all our contingency measures would be positively and moderately correlated with self-esteem instability (Chabrol et al., 2006). Indeed, contingency and self-esteem instability both correspond to forms of self-esteem fragility and are positively related in the literature (*r* ranging from 0.29 to 0.44) (Jordan and Zeigler-Hill, 2013). Next, we expected to observe strong negative correlations between our contingency measures and Rosenberg's (1965) trait self-esteem because contingency, as defined by Johnson and Blom (2007), would result from low self-esteem.

Concerning the discriminant validity of the two dimensions of the tool, considering the results obtained by Johnson and Blom (2007), we expected Competence Based Self-Esteem to be strongly and positively correlated with a measure of self-oriented perfectionism (Labrecque et al., 1998) and not correlated with a measure of need to belong (Vallieres and Vallerand, 1990). In contrast, we expected Relation Based Self-Esteem to be strongly and positively correlated with the need to belong and weakly or not correlated with self-oriented perfectionism.

Finally, we hypothesized that the level of stress felt by students 6 weeks after completing the first study session will be positively predicted by their initial level of self-esteem contingency.

#### 2.1.2 Scales used for psychometric testing

The following scales were administered to participants to test the psychometric properties of the French version of the Johnson and Blom scale:

***French translation of the Extrinsic Contingency Focus Scale*** (Leboeuf and Losier, 2012) measuring self-esteem contingency regarding extrinsic criteria (i.e., others' approval). This scale consisted of twenty items and participants responded on a Likert scale ranging from 1 (strongly disagree) to 5 (strongly agree).

***Self-esteem instability*** was measured using the self-esteem instability scale (Chabrol et al., 2006), consisting of 4 items and participants responded on a Likert scale ranging from 1 (strongly disagree) to 4 (strongly agree).

***Self-esteem level*** was measured using the Rosenberg Self-Esteem Scale (Vallieres and Vallerand, 1990). Participants responded on a scale from 1 (not at all agree) to 4 (completely agree).

***Self-oriented perfectionism*** was measured using the corresponding dimension of the Multidimensional Perfectionism Scale validated in French (Labrecque et al., 1998). Participants responded on a scale from 1 (totally disagree) to 7 (totally agree).

***The need to belong*** was measured using the French version of the Need to Belong Scale (Sanquirgo et al., 2012). Participants responded on a scale from 1 (not at all agree) to 5 (completely agree).

***Stress*** was measured using a French validation of the Perceived Stress Scale with 4 items (Lesage et al., 2012). Participants responded on a scale from 1 (never) to 5 (often).

The full list of scales administered to each sample, along with the psychometric criteria tested, is presented in Table 1.

**Table 1:** Summary of the materials used, the analyses conducted, and the psychometric criteria evaluated across the samples.

**Study**	**Sample**	**Scales**	**Psychometric criteria**	**Analyses**
**Study 1**	**Students sample 1 (*****N*** **=** **221)**	Extrinsic contingency focus scale (Leboeuf and Losier, 2012) Self-esteem instability scale (Chabrol et al., 2006)	Concurrent and convergent validity	EFA, correlations
**Study 2**	**Students sample 2 (*****N*** **=** **507) (two subsamples)**	Subsample 1 (*N =* 179): Rosenberg Self-Esteem Scale (Vallieres and Vallerand, 1990) Multidimensional Perfectionism Scale (Labrecque et al., 1998)	Convergent and discriminant validity	CFA, ESEM, Bifactor-CFA, Bifactor-ESEM, correlations
		Subsample 2 (*N =* 328 at *T1* and 140 at *T2*): Need to belong scale (Sanquirgo et al., 2012) Perceived stress scale (Lesage et al., 2012)	Convergent and discriminant validity Temporal stability and predictive validity	
**Study 2**	**Jobseekers (*****N*** **=** **270)**	Multidimensional Perfectionism Scale (Labrecque et al., 1998) Need to Belong Scale (Sanquirgo et al., 2012)	Convergent and discriminant validity	CFA, ESEM, Bifactor-CFA, Bifactor-ESEM, correlations
**Study 2**	**Employees (*****N*** **=** **328)**	Multidimensional perfectionism scale (Labrecque et al., 1998) Need to belong scale (Sanquirgo et al., 2012)	Convergent and discriminant validity	CFA, ESEM, Bifactor-CFA, Bifactor-ESEM, correlations

## 3 Study 1: exploratory factor structure and examination of concurrent and convergent validity

### 3.1 Participants and procedure

A total of 221 students participated in this study by completing an online questionnaire *via* “LimeSurvey” after a lecture. Participation was voluntary, and informed consent was obtained in writing. Participants were provided with prior information about the study objectives. Data collection took place from February 28, 2023, to May 8, 2023. The sample consisted of 185 women, 32 men, and 4 individuals identifying as other, with ages ranging from 18 to 61 years (*M* = 19.4; *SD* = 3.71). Participants were predominantly from the psychology program (84 first-year students and 92 second-year students), with a smaller proportion from health sciences (*N* = 30) and other programs, including computer science, life sciences, sports sciences (STAPS), and art (*N* = 15).

### 3.2 Results

#### 3.2.1 Exploratory structure of the scale

Prior to testing the scale's structure, inter-item correlations were analyzed to eliminate potentially redundant items (*r* > 0.70). We identified that Item 10 (“My self-esteem fluctuates easily with signs of acceptance and rejection from others”) and Item 11 (“I am sensitive to signs of dislike and rejection from others”) exceeded the redundancy threshold. To improve content validity, Item 10 was removed as it conflated two opposing situations—self-esteem dependence on acceptance vs. rejection—potentially hindering clarity.

An exploratory factor analysis was conducted using maximum likelihood estimation with oblimin rotation in Jamovi. Bartlett's test of sphericity (χ^*2*^ = 1,172, *df* = 78, *p* < 0.001) confirmed that there were adequate correlations among scale items, and the Kaiser-Meyer-Olkin (KMO) measure indicated good sampling adequacy (KMO = 0.857).

The results revealed two factors with eigenvalues > 1, explaining 46.5% of the total variance. These factors corresponded to Competence Based Self-Esteem (CBSE) and Relation Based Self-Esteem (RBSE). Most items constituting CBSE exhibited high loadings (>0.59), except for two items (3 and 4) with lower loadings (0.31 to 0.39). This factor explained 27.4% of the variance.

The second factor, RBSE, comprised the remaining items, each with loadings exceeding 0.63, and explained 19.2% of the total variance. A second analysis, after removing items 3 and 4, revealed two factors, explaining 29.4% and 21.5% of the variance, for a total of 51% of the entire scale's variance (see Table 1). McDonald's omega values showed good internal consistency for the overall contingency (ω = 0.85), CBSE (ω = 0.85), and RBSE (ω = 0.84).

#### 3.2.2 Correlation with other constructs

To test the concurrent and convergent validity of our scale, we examined its correlations with Leboeuf and Losier's (2012) scale and the self-esteem instability scale (Chabrol et al., 2006). Correlational analyses revealed that measures of self-esteem contingency strongly and positively correlated with Leboeuf and Losier's scale (*r* = 0.66, *p* < 0.001 for the overall score, *r* = 0.54, *p* < 0.001 for CBSE, and *r* = 0.55, p < 0.001 for RBSE). In line with the literature, these measures of contingency also moderately and positively correlated with self-esteem instability (*r* = 0.36, *p* < 0.001; *r* = 0.38, *p* < 0.001; *r* = 0.22, *p* < 0.001).

### 3.3 Conclusion

This first study, conducted with a student sample, allowed us to identify the two dimensions of our self-esteem contingency scale and led to the removal of one redundant item and two items with low factor loadings. As expected, the overall scale and its two dimensions—Competence Based Self-Esteem (CBSE), and Relation Based Self-Esteem (RBSE)—demonstrated good internal consistency as well as strong concurrent and convergent validity. To further strengthen these initial findings, we will conduct additional analyses in Study 2, including Confirmatory Factor Analysis (CFA), Exploratory Structural Equation Modeling (ESEM), bifactorial CFA, and bifactorial ESEM. These analyses will test the presence of a general factor corresponding to global self-esteem contingency, in addition to the two specific factors corresponding to CBSE and RBSE.

## 4 Study 2: confirmatory structure and psychometrics' qualities of the two-dimensional scale

### 4.1 Objective

The objective of this second study is multifaceted. Firstly, it aims to confirm the bidimensional structure of the scale with a new sample of students and to extend these results to unemployed individuals as well as employees. For each population, we will test different models (i.e., CFA, ESEM, Bifactor-CFA, and Bifactor-ESEM), hypothesizing that the best fit will be found among the bifactor models. These models imply that self-esteem contingency relies on a global factor while also incorporating the specific contingency upon competence and relationships. The comparison of these models will help determine the most appropriate structure to represent self-esteem contingency across these different populations. We will then test the psychometric qualities of the tool by analyzing its correlations with other constructs.

### 4.2 Method

#### 4.2.1 Participants and procedure

##### 4.2.1.1 Student sample

All students who agreed to participate in the study completed a paper-based questionnaire during their seminars. Participation was voluntary, and written consent was obtained after they were informed of the study's objectives. Data collection for subsample 1 took place from February 28, 2023, to May 8, 2023, while for subsample 2, it occurred from October 9, 2023, to November 16, 2023.

A total of 507 students responded to the questionnaire. The first subsample consisted of 179 students (149 women, 28 men) enrolled in the 2^nd^ year (156 students) and 4^th^ year (14 students) of psychology, as well as in the 1^st^ year of a Master's program in human resources management (9 students). The participants' ages ranged from 18 to 46 years (M = 21, SD = 3.61). The second subsample comprised 328 students (274 women, 50 men, 4 others) enrolled in the first year of psychology (235 students) and health studies (93 students). Their ages ranged from 18 to 25 years (*M* = 18.5, *SD* = 1).

##### 4.2.1.2 Job seekers sample

Participants were recruited from the National Agency for Adult Vocational Training (AFPA), with data collection taking place from January 3, 2023, to July 11, 2023. Participants were free to join and gave their written consent after being informed of the study's objectives. They completed a paper questionnaire during their training or support sessions.

A total of 270 job seekers responded to the questionnaire: 115 were in vocational training, and 153 were part of a return-to-employment support program. This sample included 86 men and 178 women, aged 18 to 62 years (*M* = 37, *SD* = 11.2). The reported unemployment period ranged from 0 to 240 months (*M* = 17, *SD* = 29). Note that some participants did not provide all the information, which explains discrepancies in the total number of participants.

##### 4.2.1.3 Employees sample

Participants were recruited via social networks (e.g., LinkedIn, Facebook, forums) and through a panel. They completed an online questionnaire hosted on the “Limesurvey” platform. Prior to participation, employees were informed about the study's objectives. Data collection took place between September 13, 2022, and June 19, 2023. A total of 328 employees participated and provided written consent. The sample consisted of 108 men and 178 women, aged between 20 and 62 years (*M* = 41, *SD* = 10.8).

The employees primarily worked in the commercial tertiary sector (49.2%) and the non-commercial tertiary sector (41.2%). The industrial sector (7.1%) and the construction sector (2.5%) were less represented. Most employees were on permanent contracts (80.7%) and worked full-time (86.1%). A smaller proportion worked on fixed-term contracts (19.3%) and part-time (13.9%).

#### 4.2.2 Measures

All measures used to test psychometrics qualities are presents in the Table 2.

**Table 2:** Factor loadings for the two dimensions of the contingency of self-esteem scale.

**Items**		**CBSE**	**RBSE**
1	I feel worthwhile only when I have performed well. Je me sens capable seulement quand je réussis.	0.769	
2	I think my worth as a person is determined by how well I succeed. Je pense que ce que je vaux dépend de mes réussites.	0.635	
3	My self-esteem is highly dependent upon the results of my daily actions. Ce que je pense de moi dépend beaucoup de ce que j'arrive ou non à réaliser au quotidien.	0.574	
4	No matter how well I have done a task, there is always a nagging feeling that I should have done better. Même quand je réussis quelque chose, j'ai toujours le sentiment que j'aurais pu faire mieux.	0.591	
5	When I have failed in an exam or in another context performed worse than I expected it has made me doubt my self-worth. Je doute de ma valeur quand j'échoue à un examen/test ou quand j'ai moins bien réussi que prévu.	0.752	
6	It is hard for me to forgive myself when I fail in an important task. C'est difficile pour moi de me pardonner quand j'échoue à quelque chose d'important.	0.675	
7	My feeling is that no matter how I work I'll never reach my best performance goals. J'ai l'impression que quoi que je fasse, je n'arriverai jamais à être aussi compétent que je souhaiterais l'être.	0.685	
8	I am sensitive to signs of dislike and rejection from others. Je suis sensible aux signes d'hostilité et de rejet de la part des autres.		0.639
9	It is important for my self-esteem to be loved. Être aimé est important pour que je puisse me considérer comme une bonne personne.		0.783
10	Love and support from other people makes me like myself more. L'amour et le soutien des autres me permettent de m'apprécier davantage.		0.827
11	My self-esteem strengthens considerably when others seek my company. Je me sens revalorisé quand les autres recherchent ma compagnie.		0.743
% variance		29.4	21.4

CBSE refers to Competence Based Self-Esteem; RBSE refers to Relation Based Self-Esteem. Items 1–3 correspond to the “Contingent Upon Competence” subdimension, and items 4–7 correspond to the “Self-Critical” subdimension.

#### 4.2.3 Statistical procedures of structural analysis

**Figure 1:**
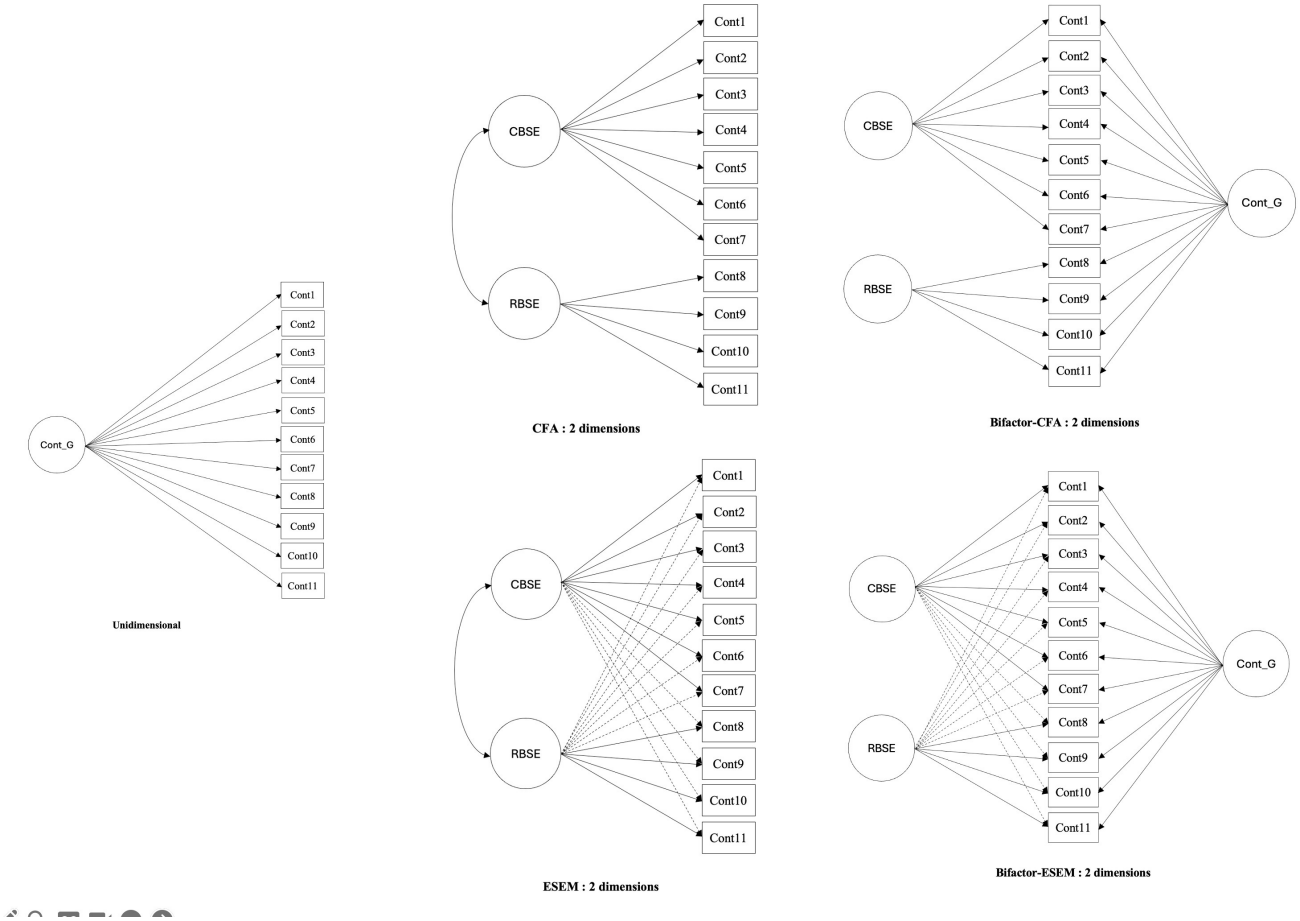
Structural diagrams of Confirmatory Factor Analysis (CFA), Exploratory Structural Equation Modeling (ESEM), Bifactor-CFA, and Bifactor-ESEM models of the two-dimensional contingent self-esteem scale. CBSE, Competence Based Self-Esteem; RBSE, Relation Based Self-Esteem. Cont_G, Global Self-Esteem Contingency.

Following the recommendations of Morin et al. (2020) and the decision tree proposed by Alamer (2022), we applied a sequential approach to compare five models. First, we tested the fit of the unidimensional model (see Figure 1). Next, we compared the two-dimensional CFA and ESEM models (i.e., CBSE, RBSE) to assess the fit of the self-esteem contingency scale. For each sample, ESEM analyses were conducted using a confirmatory rotation approach (i.e., target rotation). This method allows for pre-specifying indicators for each factor while freely estimating cross-loadings, with the aim of keeping them as close to zero as possible (Asparouhov and Muthén, 2009).

According to the guidelines of Morin et al. (2020), the ESEM solution would be preferred over the CFA when the following conditions are met: (1) improvement in fit indices, (2) reduction of factor correlations, (3) small to moderate cross-loadings that can be easily justified, and (4) adequate definition of the factors, particularly the general factor.

Next, since self-esteem contingency can be conceptualized as a global construct (Deci and Ryan, 1995; Kernis, 2003), we compared the selected CFA or ESEM solution with a corresponding bifactor solution (bifactor-CFA or bifactor-ESEM). According to Morin et al. (2020), a bifactor representation should be prioritized when: (1) the model fit improves significantly, (2) a well-defined general factor (G-factor) emerges, and (3) at least some specific factors (S-factors) are adequately defined.

All analyses were conducted with Mplus version 8.11 (Muthén and Muthén, 1998-2017). The analyses were conducted with maximum likelihood.

To assess model fit, we followed standard interpretation guidelines (Marsh et al., 2004) and used the following indices: chi-square statistic, root mean square error of approximation (RMSEA) with its 90% confidence interval, comparative fit index (CFI), Tucker-Lewis Index (TLI), and standardized root mean square residual (SRMR). For excellent model fit, RMSEA should be below 0.06, and CFI and TLI should exceed 0.95. For an acceptable fit, RMSEA should remain below 0.08, and CFI and TLI should be above 0.90. The SRMR should fall within the range of 0 to 0.08. In all models, items with significant factor loadings >0.3 were considered relevant to the corresponding factor.

### 4.3 Results

#### 4.3.1 Confirmatory structure analysis: CFA, ESEM, bifactor-CFA, and bifactor-ESEM results

The results of CFA, Bifactor-CFA, ESEM, and Bifactor-ESEM results are reported in Table 3. The results indicated poor fit to the data in the unidimensional model, for each population.

**Table 3:** Fit indices for the unidimensional and two-dimensional models of the contingent self-esteem scale.

**Sample**	**Model**	***χ2***	***df***	***p***	**RMSEA**	**90% *CI***	**CFI**	**TLI**	**SRMR**
Students (*N* = 507)	**Unidimensional**	682	44	0.000	0.169	[0.158, 0.180]	0.642	0.552	0.122
	**CFA**	149	43	0.000	0.070	[0.058, 0.082]	0.940	0.924	0.049
	**ESEM**	109.826	34	0.000	0.066	[0.053, 0.080]	0.957	0.931	0.034
	**Bifactor-CFA**	53.574	33	<0.05	0.037	[0.019, 0.053]	0.987	0.979	0.023
	**Bifactor-ESEM**	46.864	25	<0.01	0.042	[0.022, 0.060]	0.988	0.973	0.018
Jobseekers (*N* = 270)	**Unidimensional**	276	44	0.000	0.140	[0.124, 0.156]	0.774	0.717	0.094
	**CFA**	99.3	43	0.000	0.070	[0.052, 0.088]	0.945	0.930	0.053
	**ESEM**	71.734	34	<0.01	0.064	[0.043, 0.085]	0.963	0.940	0.034
	**Bifactor-CFA**	25.265	33	ns	0.000	[0.000, 0.027]	1.000	1.000	0.023
	**Bifactor-ESEM**	15.334	25	ns	0.000	[0.000, 0.013]	1.000	1.000	0.014
Employees (*N* = 328)	**Unidimensional**	545	44	0.000	0.186	[0.173, 0.201]	0.601	0.501	0.119
	**CFA**	219	43	0.000	0.112	[0.097, 0.127]	0.859	0.820	0.072
	**ESEM**	173.073	34	0.000	0.112	[0.095, 0.128]	0.889	0.820	0.052
	**Bifactor-CFA**	89.598	33	0.000	0.072	[0.055, 0.090]	0.955	0.924	0.043
	**Bifactor-ESEM**	63.105	25	0.000	0.068	[0.047, 0.089]	0.969	0.933	0.026

##### 4.3.1.1 CFA vs. ESEM - 2 dimensions

The results of our analyses indicate that the unidimensional model does not exhibit good fit indices across all populations (CFI and TLI < 0.780; RMSEA and SRMR > 0.094). In contrast, the two-dimensional CFA and ESEM models (i.e., CBSE and RBSE) display acceptable fit indices for students and job seekers (CFI and TLI > 0.923; RMSEA < 0.071; SRMR < 0.054). Compared to the CFA model, the ESEM model shows better fit indices for both students (ΔCFI = +0.027; ΔTLI = +0.007; ΔRMSEA = −0.004; ΔSRMR = −0.015) and job seekers (ΔCFI = +0.018; ΔTLI = +0.010; ΔRMSEA = −0.006; ΔSRMR = −0.019). A similar pattern is observed among employees, although the fit indices do not meet acceptability criteria (see Table 4). Analyzing the factor loadings in the CFA model for each population, we observe that items 1 to 7 load significantly (0.344 < λ < 0.775) on the CBSE dimension, while items 8 to 11 load significantly on the RBSE dimension (0.623 < λ < 0.814). Similar results are found in the ESEM model, which also includes low cross-loadings (λ < 0.240). Comparing the factor correlations in the ESEM and CFA models, we find that correlations are consistently lower in the ESEM model than in the CFA model (−0.026 < Δ*r* < −0.030), further supporting the ESEM model (Morin et al., 2020).

**Table 4:** Standardized factor loadings (λ) from the CFA, ESEM, bifactor-CFA and bifactor-ESEM models of the two-dimensional contingent self-esteem scale.

	**CFA**		**ESEM**		**Bifactor-CFA**			**Bifactor-ESEM**		
**Items**	**F1 (**λ**)**	**F2 (**λ**)**	**F1 (**λ**)**	**F2 (**λ**)**	**GF (**λ**)**	**F1 (**λ**)**	**F2 (**λ**)**	**GF (**λ**)**	**F1 (**λ**)**	**F2 (**λ**)**
**Students**										
1	**0.692** ^***^		**0.697** ^***^	−0.015	**0.686** ^***^	0.139		**0.695** ^***^	−0.071	−0.015
2	**0.752** ^***^		**0.782** ^***^	−0.054	**0.775** ^***^	0.244		**0.796** ^***^	−0.162	**−0.063** ^*^
3	**0.675** ^***^		**0.672** ^***^	0.015	**0.676** ^***^	0.170		**0.687** ^***^	−0.108	0.013
4	**0.345** ^***^		**0.343** ^***^	−0.013	**0.356** ^***^	**−0.367** ^**^		**0.324** ^***^	**0.435** ^**^	−0.019
5	**0.717** ^***^		**0.701** ^***^	0.035	**0.715** ^***^	−0.119		**0.698** ^***^	0.162	0.034
6	**0.584** ^***^		**0.552** ^***^	0.065	**0.592** ^***^	**−0.251** ^*^		**0.565** ^***^	**0.291** ^**^	0.059
7	**0.554** ^***^		**0.549** ^***^	0.006	**0.564** ^***^	**−0.263** ^*^		**0.539** ^***^	**0.319** ^**^	0.002
8		**0.737** ^***^	**0.140** ^***^	**0.672** ^***^	**0.367** ^***^		**0.633** ^***^	**0.375** ^***^	−0.043	**0.633** ^***^
9		**0.797** ^***^	**0.074** ^*^	**0.755** ^***^	**0.334** ^***^		**0.710** ^***^	**0.338** ^***^	0.019	**0.707** ^***^
10		**0.715** ^***^	**−0.140** ^***^	**0.799** ^***^	**0.134** ^**^		**0.748** ^***^	**0.138** ^**^	0.019	**0.748** ^***^
11		**0.658** ^***^	−0.050	**0.687** ^***^	**0.188** ^***^		**0.645** ^***^	**0.191** ^***^	0.038	**0.642** ^***^
**Job seekers**										
1	**0.674** ^***^		**0.717** ^***^	−0.064	**0.801** ^***^	**0.350** ^*^		**0.637** ^***^	**0.289** ^*^	−0.035
2	**0.686** ^***^		**0.726** ^***^	−0.062	**0.830** ^***^	**0.566** ^**^		**0.640** ^***^	**0.505** ^*^	−0.030
3	**0.774** ^***^		**0.745** ^***^	0.060	**0.898** ^***^	**0.374** ^**^		**0.724** ^***^	**0.316** ^*^	0.075
4	**0.480** ^***^		**0.511** ^***^	−0.057	**0.572** ^***^	−0.100		**0.507** ^***^	−0.061	−0.076
5	**0.724** ^***^		**0.700** ^***^	0.035	**0.894** ^***^	−0.056		**0.738** ^***^	−0.021	0.006
6	**0.642** ^***^		**0.608** ^***^	0.062	**0.829** ^***^	−0.140		**0.670** ^***^	−0.098	0.025
7	**0.669** ^***^		**0.620** ^***^	0.079	**0.984** ^***^	−0.367		**0.748** ^***^	−0.238	0.007
8		**0.624** ^***^	**0.181** ^**^	**0.514** ^***^	**0.579** ^***^		**0.568** ^***^	**0.444** ^***^	−0.064	**0.439** ^***^
9		**0.700** ^***^	**0.134** ^*^	**0.607** ^***^	**0.553** ^***^		**0.688** ^***^	**0.415** ^***^	**0.103** ^*^	**0.540** ^***^
10		**0.761** ^***^	**−0.135** ^**^	**0.873** ^***^	**0.359** ^***^		**0.905** ^***^	**0.295** ^***^	0.002	**0.763** ^***^
11		**0.701** ^***^	−0.060	**0.738** ^***^	**0.364** ^***^		**0.741** ^***^	**0.313** ^***^	−0.048	**0.635** ^***^
**Employees**										
1	**0.631** ^***^		**0.652** ^***^	−0.048	**0.591** ^***^	**−0.271** ^**^		**0.611** ^***^	**0.227** ^*^	−0.045
2	**0.638** ^***^		**0.631** ^***^	−0.007	**0.619** ^***^	**−0.639** ^***^		**0.669** ^***^	**0.583** ^***^	−0.046
3	**0.654** ^***^		**0.551** ^***^	**0.178** ^***^	**0.600** ^***^	**−0.336** ^***^		**0.611** ^***^	**0.316** ^***^	**0.160** ^***^
4	**0.482** ^***^		**0.545** ^***^	−0.091	**0.521** ^***^	0.150		**0.531** ^***^	−0.223	**−0.094** ^*^
5	**0.679** ^***^		**0.652** ^***^	0.056	**0.722** ^***^	0.186		**0.690** ^***^	**−0.221** ^*^	0.049
6	**0.607** ^***^		**0.594** ^***^	0.047	**0.659** ^***^	0.185		**0.631** ^***^	**−0.217** ^*^	0.039
7	**0.551** ^***^		**0.610** ^***^	−0.077	**0.591** ^***^	0.133		**0.601** ^***^	−0.206	−0.080
8		**0.635** ^***^	**0.238** ^***^	**0.521** ^***^	**0.453** ^***^		**0.476** ^***^	**0.453** ^***^	−0.051	**0.475** ^***^
9		**0.812** ^***^	0.052	**0.769** ^***^	**0.361** ^***^		**0.708** ^***^	**0.362** ^***^	0.021	**0.701** ^***^
10		**0.813** ^***^	**−0.117** ^***^	**0.892** ^***^	**0.256** ^***^		**0.806** ^***^	**0.252** ^***^	−0.008	**0.813** ^***^
11		**0.734** ^***^	−0.050	**0.762** ^***^	**0.260** ^***^		**0.695** ^***^	**0.258** ^***^	0.068	**0.697** ^***^

GF, global factor of self-esteem contingency; F1, Contingent upon competence; F2, Relation Based Self-Esteem (RBSE). ^*^p < 0.05; ^**^p < 0.01; ^***^p < 0.001.

##### 4.3.1.2 Bifactor-CFA and bifactor-ESEM – 2 dimensions

The bifactor-CFA model shows good fit indices for students and job seekers (CFI and TLI > 0.978; RMSEA < 0.038; SRMR = 0.023). For employees, the fit indices are acceptable (CFI and TLI > 0.923; RMSEA = 0.071; SRMR = 0.043). Compared to the CFA and ESEM models, the bifactor-CFA model consistently demonstrates better fit. Additionally, the bifactor-ESEM model offers improved fit indices compared to the bifactor-CFA model for employees (ΔCFI = +0.014; ΔTLI = +0.009; ΔRMSEA = −0.004; ΔSRMR = −0.017) and, to a lesser extent, for job seekers (ΔSRMR = −0.009). However, the results are more mixed for students, as only the SRMR index is more favorable for the bifactor-ESEM model compared to the bifactor-CFA model (ΔCFI = −0.001; ΔTLI = −0.006; ΔRMSEA = +0.005; ΔSRMR = −0.005). Analyzing the loadings associated with the bifactor-CFA models, we observe that all items load significantly onto the global factor. Notably, items 10 and 11 display relatively low loadings for students and employees (0.133 < λ < 0.261). Regarding the specific factors, all items belonging to the RBSE dimension exhibit high and significant loadings on this factor (0.475 < λ < 0.906). More nuanced results are observed with the CBSE dimension. Specifically, only a few items load significantly onto this dimension, and these items vary across populations. Items 1, 2, and 3 load significantly for job seekers and employees, while items 4, 6, and 7 do so for students. Additionally, we observe that the loadings for items 1 to 3 are systematically opposite in sign to those of items 4 to 7, a pattern consistent across all bifactor-ESEM models. These *two* groups of items correspond to the *two* subdimensions of CBSE initially described by Johnson and Blom (2007): contingent upon competence (items 1 to 3) and self-critical (items 4 to 7). While the distinction between these *two* subdimensions did not emerge in the exploratory factor analyses previously conducted (using the eigenvalue >1 criterion), it appears that considering them may be necessary for a better understanding of the scale. In summary, although the bifactor-CFA model offers an improvement in fit indices and includes a well-defined general contingency factor as well as a specific factor adequately capturing RBSE (Morin et al., 2020), we believe this model warrants comparison with alternative three-factor solutions to clarify the unexpected loadings observed on the CBSE factor.

**Figure 2:**
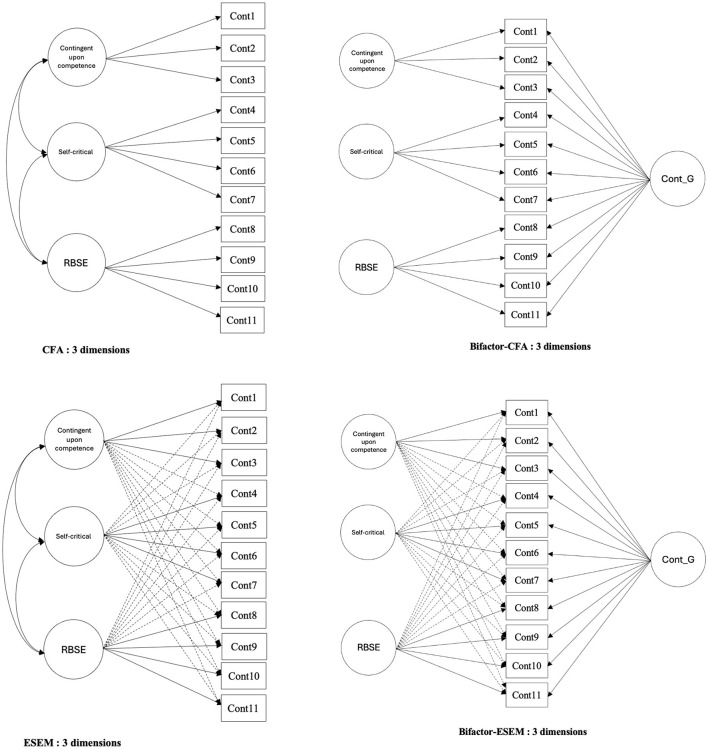
Structural diagrams of confirmatory factor analysis (CFA), exploratory structural equation modeling (ESEM), bifactor-CFA, and bifactor-ESEM models of the three-dimensional contingent self-esteem scale.

Given this unexpected result, we tested the fit of four new models (i.e., CFA, ESEM, Bifactor-CFA, and Bifactor-ESEM) representing contingent upon competence, self-critical, and Relation Based Self-Esteem (see Figure 2). We subsequently compared the fit of each of these models to their two-factor equivalents.

##### 4.3.1.3 CFA vs. ESEM – 3 dimensions

Regarding the fit quality of the three-factor models, the results (see Table 5) indicate that the CFA models (i.e., contingent upon competence, self-critical, and RBSE) show good fit indices for students and job seekers (CFI and TLI > 0.951; RMSEA < 0.056; and SRMR < 0.044) and acceptable fit indices for employees (CFI = 0.929; TLI = 0.905; RMSEA = 0.081; SRMR = 0.058). Compared to the CFA model, the ESEM model demonstrates better fit indices across all populations: for students (ΔCFI = +0.024; ΔTLI = +0.021; ΔRMSEA = −0.013; ΔSRMR = −0.025), for job seekers (ΔCFI = +0.015; ΔTLI = +0.020; ΔRMSEA = −0.037; ΔSRMR = −0.023), and for employees (ΔCFI = +0.040; ΔTLI = +0.028; ΔRMSEA = −0.013; ΔSRMR = −0.032).

**Table 5:** Fit indices for the three-dimensional models of the contingent self-esteem scale.

**Sample**	**Model**	***χ2***	***df***	***p***	**RMSEA**	**90% *CI***	**CFI**	**TLI**	**SRMR**
Students (*N* = 507)	**CFA**	105	41	0.000	0.055	[0.042, 0.069]	0.964	0.952	0.043
	**ESEM**	46.864	25	<0.05	0.042	[0.022, 0.060]	0.988	0.973	0.018
	**Bifactor CFA**	43.933	33	ns	0.026	[0.000, 0.044]	0.994	0.990	0.019
	**Bifactor ESEM**	28	17	<0.05	0.037	[0.010, 0.060]	0.993	0.978	0.013
Jobseekers (*N* = 270)	**CFA**	56.1	41	0.058	0.037	[0.000, 0.059]	0.985	0.980	0.037
	**ESEM**	15.334	25	ns	0.000	[0.000, 0.013]	1.000	1.000	0.014
	**Bifactor CFA**	23.958	33	ns	0.000	[0.000, 0.023]	1.000	1.000	0.023
	**Bifactor ESEM**	5.92	17	ns	0.000	[0.000, 0.000]	1.000	1.000	0.009
Employees (*N* = 328)	**CFA**	130	41	0.000	0.081	[0.066, 0.097]	0.929	0.905	0.058
	**ESEM**	63.105	25	0.000	0.068	[0.047, 0.089]	0.969	0.933	0.026
	**Bifactor CFA**	65.003	33	<0.01	0.054	[0.029, 0.070]	0.974	0.957	0.038
	**Bifactor ESEM**	27.555	17	ns	0.044	[0.000, 0.072]	0.992	0.973	0.016

Analyzing the factor loadings of the CFA model for each population (see Table 6), we observe that items 1 to 3 load significantly (0.698 < λ < 0.825) on the “contingent upon competence” dimension, items 4 to 7 load significantly (0.391 < λ < 0.765) on the “self-critical” dimension, and items 8 to 11 load significantly on the RBSE dimension (0.624 < λ < 0.814). Similar results are found with the ESEM model, which also includes minor cross-loadings (λ < 0.320). Comparing the factor correlations of the three-dimensional ESEM and CFA models, we find that the correlations are consistently lower in the ESEM models than in the CFA models (−0.030 < Δr < −0.131), which supports the ESEM model (Morin et al., 2020).

**Table 6:** Standardized factor loadings (λ) from the CFA, ESEM, Bifactor-CFA and bifactor-ESEM models of the three-dimensional contingent self-esteem scale.

	**CFA**			**ESEM**			**Bifactor-CFA**				**Bifactor-ESEM**			
	**F1 (**λ**)**	**F2 (**λ**)**	**F3 (**λ**)**	**F1 (**λ**)**	**F2 (**λ**)**	**F3 (**λ**)**	**GF (**λ**)**	**F1 (**λ**)**	**F2 (**λ**)**	**F3 (**λ**)**	**GF (**λ**)**	**F1 (**λ**)**	**F2 (**λ**)**	**F3 (**λ**)**
**Students**														
1	**0.709** ^***^			**0.598** ^***^	0.127	0.020	**0.633** ^***^	**0.270** ^**^			**0.555** ^***^	**0.430** ^***^	**0.088** ^*^	0.027
2	**0.802** ^***^			**0.719** ^***^	0.041	−0.016	**0.672** ^***^	**0.548** ^***^			**0.640** ^***^	**0.504** ^***^	−0.046	−0.021
3	**0.699** ^***^			**0.627** ^***^	0.070	0.053	**0.611** ^***^	**0.297** ^***^			**0.566** ^***^	**0.401** ^***^	−0.030	0.044
4		**0.392** ^***^		**−0.255** ^***^	**0.701** ^***^	−0.056	**0.280** ^***^		**0.781** ^*^		**0.413** ^***^	−0.122	0.114	−0.050
5		**0.764** ^***^		**0.320** ^***^	**0.440** ^***^	0.046	**0.760** ^***^		0.076		**0.697** ^***^	**0.167** ^*^	−0.022	0.023
6		**0.638** ^***^		0.066	**0.571** ^***^	0.052	**0.597** ^***^		0.173		**0.731** ^***^	**−0.139** ^*^	−0.092	−0.012
7		**0.581** ^***^		0.032	**0.608** ^***^	−0.012	**0.546** ^***^		0.240		**0.610** ^***^	0.010	**0.789** ^***^	−0.021
8			**0.737** ^***^	**0.151** ^**^	−0.027	**0.691** ^***^	**0.399** ^***^			**0.618** ^***^	**0.347** ^***^	**0.111** ^**^	0.000	**0.641** ^***^
9			**0.797** ^***^	0.032	0.036	**0.760** ^***^	**0.356** ^***^			**0.698** ^***^	**0.341** ^***^	0.035	0.017	**0.705** ^***^
10			**0.715** ^***^	**−0.128** ^*^	−0.034	**0.796** ^***^	**0.155** ^**^			**0.744** ^***^	**0.173** ^**^	−0.070	−0.037	**0.737** ^***^
11			**0.658** ^***^	−0.080	0.024	**0.683** ^***^	**0.192** ^***^			**0.646** ^***^	**0.220** ^***^	−0.041	−0.011	**0.632** ^***^
**Job seekers**														
1	**0.704** ^***^			**0.595** ^***^	0.150	−0.022	**0.631** ^***^	**0.293** ^*^			**0.626** ^***^	**0.287** ^*^	0.037	−0.050
2	**0.744** ^***^			**0.894** ^***^	−0.107	−0.018	**0.608** ^***^	**0.587** ^***^			**0.583** ^***^	**0.651** ^***^	0.045	−0.015
3	**0.824** ^***^			**0.640** ^***^	0.135	**0.106** ^*^	**0.753** ^***^	**0.273** ^*^			**0.765** ^***^	**0.270** ^*^	−0.057	0.030
4		**0.511** ^***^		0.079	**0.486** ^***^	−0.070	**0.446** ^***^		0.224		**0.421** ^***^	0.082	**0.295** ^*^	−0.047
5		**0.760** ^***^		**0.189** ^*^	**0.577** ^***^	0.029	**0.702** ^***^		**0.230** ^*^		**0.661** ^***^	0.111	**0.303** ^***^	0.023
6		**0.675** ^***^		0.060	**0.609** ^***^	0.049	**0.641** ^***^		0.202		**0.674** ^***^	−0.068	0.168	−0.005
7		**0.739** ^***^		−0.108	**0.843** ^***^	0.032	**0.624** ^***^		**0.574** ^*^		**0.642** ^***^	−0.044	**0.490** ^**^	0.039
8			**0.625** ^***^	−0.028	**0.218** ^*^	**0.512** ^***^	**0.472** ^***^			**0.409** ^***^	**0.514** ^***^	−0.132	−0.039	**0.395** ^***^
9			**0.699** ^***^	**0.177** ^*^	−0.046	**0.625** ^***^	**0.455** ^***^			**0.511** ^***^	**0.428** ^***^	0.103	0.001	**0.527** ^***^
10			**0.761** ^***^	−0.035	−0.112	**0.874** ^***^	**0.319** ^***^			**0.759** ^***^	**0.312** ^**^	−0.005	0.005	**0.764** ^***^
11			**0.702** ^***^	−0.077	0.016	**0.731** ^***^	**0.329** ^***^			**0.626** ^***^	**0.326** ^***^	−0.035	0.036	**0.626** ^***^
**Employees**														
1	**0.665** ^***^			**0.542** ^***^	**0.191** ^**^	**−0.049**	**0.527** ^***^	**0.362** ^***^			**0.488** ^***^	**0.431** ^***^	0.007	−0.005
2	**0.774** ^***^			**0.981** ^***^	**−0.132** ^**^	**−0.074** ^**^	**0.460** ^***^	**0.830** ^***^			**0.442** ^***^	**0.755** ^***^	**0.066** ^*^	−0.031
3	**0.739** ^***^			**0.602** ^***^	0.041	**0.168** ^***^	**0.548** ^***^	**0.402** ^***^			**0.471** ^***^	**0.498** ^***^	**−0.084** ^*^	**0.200** ^*^
4		**0.550** ^***^		−0.006	**0.606** ^***^	−0.070	**0.419** ^***^		**0.617** ^*^		**0.564** ^***^	−0.037	**0.305** ^*^	−0.083
5		**0.742** ^***^		0.036	**0.669** ^***^	**0.089** ^*^	**0.748** ^***^		0.089		**0.885** ^***^	−0.062	−0.451^***^	−0.046
6		**0.678** ^***^		0.017	**0.629** ^***^	0.076	**0.671** ^***^		0.089		**0.614** ^***^	0.038	0.007	0.048
7		**0.616** ^***^		0.042	**0.632** ^***^	−0.055	**0.513** ^***^		0.447		**0.651** ^***^	−0.017	**0.354** ^*^	−0.074
8			**0.635** ^***^	0.039	**0.219** ^***^	**0.531**	**0.486** ^***^			**0.452** ^***^	**0.424** ^***^	0.066	0.060	**0.490** ^***^
9			**0.812** ^***^	0.035	0.021	**0.769**	**0.385** ^***^			**0.695** ^***^	**0.351** ^***^	0.059	0.012	**0.708** ^***^
10			**0.813** ^***^	−0.071	−0.055	**0.889**	**0.295** ^***^			**0.791** ^***^	**0.262** ^***^	−0.015	**−0.108** ^**^	**0.804** ^***^
11			**0.734** ^***^	0.044	−0.095	**0.758**	**0.280** ^***^			**0.689** ^***^	**0.221** ^***^	**0.081** ^*^	0.026	**0.716** ^***^

GF, global factor of Self-Esteem Contingency; F1, Contingent upon competence; F2, self-critical; F3, Relation Based Self-Esteem (RBSE). ^*^p < 0.05; ^**^p < 0.01; ^***^p < 0.001.

##### 4.3.1.4 Bifactor-CFA and bifactor-ESEM−3 dimensions

The bifactor-CFA models show good fit indices for all populations (CFI and TLI > 0.956; RMSEA < 0.055; SRMR < 0.039). Like the two-factor models, the bifactor-CFA model with three factors exhibits better fit compared to CFA and ESEM models, particularly for students (ΔCFI = +0.006; ΔTLI = +0.017; ΔRMSEA = −0.016; ΔSRMR = +0.001) and employees (ΔCFI = +0.005; ΔTLI = +0.024; ΔRMSEA = −0.014; ΔSRMR = +0.012). For job seekers, differences are minimal (ΔSRMR = +0.009), likely due to the already strong fit of the ESEM model. The bifactor-ESEM model provides better fit than the bifactor-CFA model, but only for employees (ΔCFI = +0.018; ΔTLI = +0.016; ΔRMSEA = −0.010; ΔSRMR = −0.018). For students, the bifactor-ESEM model fits less well than its CFA counterpart (ΔCFI = −0.001; ΔTLI = −0.012; ΔRMSEA = +0.011; ΔSRMR = −0.006). For job seekers, the differences between bifactor-ESEM, ESEM, and bifactor-CFA models are negligible. During the estimation process, we encountered convergence difficulties for the bifactor-ESEM model among students and employees. To address this, we reduced the convergence criterion to 0.01. While this allowed the model to converge, additional issues arose, such as parameter estimates exceeding 1 and negative residual variances. To resolve these, we constrained improper parameter estimates to acceptable values following the recommendations of Swami et al. (2023). Comparing all three-factor models with their two-factor counterparts reveals that the three-factor models generally fit the data better. This improvement is particularly evident for students and employees. For job seekers, however, the two-factor model already demonstrated a saturated fit, leading to minimal additional benefit from the three-factor structure. The bifactor-CFA model achieves strong fit across populations, and the bifactor-ESEM model provides marginal improvements in some cases, particularly for employees. However, convergence and parameter issues with bifactor-ESEM models suggest that their applicability requires careful consideration. Further research could explore alternative parameterization or modifications to improve fit and stability for challenging populations like students. When analyzing the loadings of the three-factor bifactor CFA models, we observe that, across all populations, all items load significantly onto the general factor (G) (0.154 < λ < 0.761), as well as onto the specific factors related to contingent upon competence (F1; 0.269 < λ < 0.831) and RBSE (F3; 0.408 < λ < 0.792). However, while the four items related to self-criticism (items 4 to 7) contribute to explaining the general factor (0.279 < λ < 0.761), they do not necessarily load significantly onto the specific factor (F2) corresponding to self-criticism. Specifically, only item 4 for employees (λ = 0.617) and students (λ = 0.781), as well as items 5 and 7 for job seekers (λ = 0.230; λ = 0.574), load significantly onto this factor. The Bifactor-ESEM models also reveal that the estimated parameter scores highlight the presence of a general factor (G) (0.172 < λ < 0.732), as well as two well-defined specific factors corresponding to “contingent upon competence” (0.286 < λ < 0.756) and RBSE (0.394 < λ < 0.805). Like the bifactor CFA model, while the items associated with self-criticism contribute to the general factor, they do not fully capture the specificity of the self-criticism factor compared to the other two factors. Moreover, one item related to this factor (i.e., item 5) loads significantly and negatively onto the self-criticism factor (λ = −0.451). It is likely that the constraints we imposed to make the model operational are the cause of this result.

#### 4.3.2 Conclusion

Following the recommendations of Morin et al. (2020), we can conclude that the most relevant model is the three-dimensional bifactor-CFA model. This model shows better fit indices than the CFA and ESEM models, a well-defined global self-esteem contingency factor, two well-defined specific factors, and a specific factor (self-critical) that contributes primarily to the global factor.

### 4.4 Other psychometric criteria

#### 4.4.1 Descriptive statistics and internal consistency of dimensions of the contingent self-esteem scale

Descriptive statistics, as well as Cronbach's alpha and McDonald's omega coefficients, are presented in Table 7. Descriptively, we observe scores close to the theoretical mean of the scale across all dimensions (2.95 < *M* < 3.59). The alpha and omega coefficients suggest good internal consistency *(all* α and ω > 0.66).

**Table 7:** Descriptive statistics and internal consistency of dimensions of the contingent self-esteem scale.

**Sample**	**Variable**	***M***	***SD***	**Alpha**	**Omega**
Students (*N* = 507)	Global self-esteem contingency	3.51	0.71	0.82	0.82
	Contingent upon competence	3.41	0.97	0.78	0.78
	Self-critical	3.53	0.88	0.70	0.70
	RBSE	3.56	0.95	0.82	0.82
Students (*N* = 507) - Subsample 1 (*N* = 179)	Global self-esteem contingency	3.47	0.70	0.81	0.82
	Contingent upon competence	3.39	0.99	0.81	0.81
	Self-critical	3.48	0.88	0.67	0.68
	RBSE	3.52	0.95	0.82	0.82
Students (*N* = 507) - Subsample 2 (*N* =328)	Global self-esteem contingency	3.52	0.71	0.82	0.82
	Contingent upon competence	3.42	0.97	0.76	0.76
	Self-critical	3.55	0.88	0.71	0.72
	RBSE	3.58	0.95	0.82	0.82
Jobseekers (*N* = 270)	Global self-esteem contingency	3.11	0.79	0.85	0.86
	Contingent upon competence	2.96	1.05	0.80	0.80
	Self-critical	3.22	0.95	0.77	0.77
	RBSE	3.12	0.97	0.79	0.79
Employees (*N* = 328)	Global self-esteem contingency	3.32	0.68	0.83	0.83
	Contingent upon competence	3.13	0.93	0.77	0.77
	Self-critical	3.16	0.90	0.75	0.75
	RBSE	3.63	0.86	0.83	0.84

#### 4.4.2 Effect of gender and sample by age

An analysis of covariance (ANCOVA) was conducted to examine the effect of gender, sample, and their interaction on various measures of self-esteem contingency, controlling for age. Regarding general self-esteem contingency, a significant effect of gender was observed, with women scoring higher than men [*F*_(1,1,038)_ = 4.17, *p* = 0.041, η*^2^p* = 0.004], though the effect size was small. The sample also significantly influenced the results [*F*_(2,1,038)_ = 9.69, *p* < 0.001, η*^2^p* = 0.018], but this effect was moderate. It should be noted that the Levene's test (*p* < 0.05) indicated a violation of the homogeneity of variances assumption, suggesting these results should be interpreted cautiously. *Post hoc* analyses using the Games-Howell test, excluding age, revealed significant differences between populations, with students scoring the highest, followed by employees and unemployed individuals (differences ranging from 0.16 to 0.39, *p* < 0.001 to *p* = 0.005). However, neither age [*F*_(1,1,038)_ = 3.07, *p* = 0.080, η*^2^p* = 0.003] nor the interaction between gender and sample [*F*_(2,1,038)_ = 1.25, *p* = 0.288, η*^2^p* = 0.002] showed significant effects. For “contingent upon competence,” a significant effect of gender was also found, with women scoring higher, though the effect size was modest [*F*_(1,1,038)_ = 6.69, *p* = 0.010, η*^2^p* = 0.006]. Similarly, the sample had a significant effect [*F*_(2,1,038)_ = 3.53, *p* = 0.030, η*^2^p* = 0.007], though small in magnitude. *Post hoc* analyses using Tukey's test showed that students scored significantly higher than employees and unemployed individuals, with no significant differences between the latter two groups (differences ranging from −0.26 to −0.42, *p* < 0.001). Neither age [*F*_(1,1,038)_ = 2.80, *p* = 0.095, η*^2^p* = 0.003] nor the interaction between gender and sample [*F*_(2,1,038)_ = 2.71, *p* = 0.067, η*^2^p* = 0.005] showed significant effects, though the latter was marginally significant. Decomposing this interaction revealed one notable difference in competence-based contingency, which was higher among female students compared to male students (difference = −0.41, *p* < 0.001). Finally, for relationship-based contingency, no significant differences were observed for gender [*F*_(1,1,038)_ = 0.036, *p* = 0.850, η*^2^p* = 0.000] or age [*F*_(1,1,038)_ = 0.0361, *p* = 0.849, η*^2^p* = 0.000]. However, the sample showed a significant effect (*F*_(2,1,038)_ = 19.35, *p* < 0.001, η*^2^p* = 0.036], with marked differences between unemployed individuals and employees/students (differences ranging from −0.44 to −0.52, *p* < 0.001). In summary, while some differences were statistically significant, the moderate to small effect sizes indicate that these results should be interpreted with caution, particularly given the limitations related to variance homogeneity and the observed effect sizes.

#### 4.4.3 Convergent and discriminant validity

Given that self-criticism contributes more to the global factor than to any specific dimension, we focused our analyses on the “contingent upon competence” dimension (items 1–3) rather than the original CBSE (items 1–7) dimension (see Tables 8–10). We tested how the various constructs correlate with self-esteem levels (convergent validity) and how the “contingent upon competence” and RBSE dimensions distinctly correlate with self-oriented perfectionism and the need for belonging (discriminant validity). The results of the correlations between the different dimensions of self-esteem contingency and Rosenberg's self-esteem are consistent with our hypotheses. We found that global contingency is strongly and negatively correlated with self-esteem across all three populations (−0.63 < *r* < −0.68). The “contingent upon competence” dimension is moderately correlated with self-esteem for unemployed individuals (*r* = −0.35, *p* < 0.001) and employees (*r* = −0.40, *p* < 0.001), while it is strongly correlated with self-esteem for students (*r* = −0.55, *p* < 0.001). RBSE shows a moderate correlation with self-esteem across all three groups (−0.31 < *r* < −0.44). Notably, the “self-critical” dimension is consistently strongly and negatively correlated with self-esteem (−0.59 < *r* < −0.68). Together, these results highlight the expected relationship between self-esteem levels and contingency, with a significant role played by self-criticism in self-esteem levels. Regarding the discriminant validity of the dimensions, self-oriented perfectionism correlates significantly with the “contingent upon competence” factor. This correlation is moderate for students (*r* = 0.46, *p* < 0.001) and employees (*r* = 0.26, *p* < 0.001), while it is weak for unemployed individuals (*r* = 0.18, *p* < 0.01). In contrast, self-oriented perfectionism shows a weak correlation with RBSE for students (*r* = 0.22, *p* < 0.001) and employees (*r* = 0.14, *p* < 0.05) and does not correlate significantly with this dimension for unemployed individuals. Additionally, we tested the partial correlations between the two dimensions of self-esteem contingency and self-oriented perfectionism, controlling each dimension for the other. We observed that self-oriented perfectionism does not correlate significantly with RBSE when controlling for “contingent upon competence” (students: *r* = 0.10, *ns*; job seekers: *r* = 0.032, *ns*; employees: *r* = 0.07, *ns*). In contrast, “contingent upon competence” significantly correlates with self-oriented perfectionism when controlling for RBSE (*r* = 0.43, *p* < 0.001 for students; *r* = 0.16, *p* < 0.05 for job seekers; *r* = 0.23, *p* < 0.001 for employees). Consistent with our hypotheses, the need for belonging moderately correlates with “contingent upon competence” (0.30 < *r* < 0.42) and strongly correlates with RBSE (0.68 < *r* < 0.77). Controlling for “contingent upon competence,” the correlations between RBSE and the need for belonging remain strong (0.64 < *r* < 0.75). However, the correlations between “contingent upon competence” and the need for belonging become moderate for employees (*r* = 0.29, *p* < 0.001), weak for students (*r* = 0.17, *p* < 0.01), and non-significant for unemployed individuals (*r* = 0.11, *ns*). In summary, these results support the good discriminant validity of the two factors of the scale, further confirming the distinctiveness of the “contingent upon competence” and RBSE dimensions.

**Table 8:** Correlational matrix for the student sample (*N* = 507).

**Variable**	**1**	**2**	**3**	**4**	**5**	**6**	**7**
1. Global self-esteem contingency	_						
2. Contingent upon competence	0.77	_					
3. Self-critical	0.80	0.58	_				
4. RBSE	0.71	0.27	0.27	_			
5. Self-esteem	−0.64	−0.55	−0.60	−0.32	_		
6. Self-oriented perfectionism	0.48	0.46	0.44	0.22	−0.28	_	
7. Need to belong	0.62	0.31	0.31	0.76	_	_	_

All correlations are significant at *p* < 0.01. The correlations between Self-esteem, Self-oriented perfectionism, and the different dimensions of contingent self-esteem refer to Subsample 1 (*N* = 179). The correlations between the Need to Belong and the different dimensions of contingent self-esteem refer to Subsample 2 (*N* = 328). The correlations between all dimensions of contingent self-esteem refer to the combined samples (*N* = 507).

**Table 9:** Correlational matrix for the job seekers sample (*N* = 270).

**Variable**	**1**	**2**	**3**	**4**	**5**	**6**	**7**
1. Global self-esteem contingency	_						
2. Contingent upon competence	0.80	_					
3. Self-critical	0.84	0.62	_				
4. RBSE	0.76	0.38	0.41	_			
5. Self-esteem	−0.67	−0.35	−0.65	−0.43	_		
6. Self-oriented perfectionism	0.23	0.18	0.28	0.10	−0.07	_	
7. Need to belong	0.60	0.34	0.38	0.69	−0.39	0.17	_

All correlations are significant at *p* < 0.01 except correlation between Self-oriented perfectionism and RBSE (ns) and between Self-oriented perfectionism and Self-esteem (ns).

**Table 10:** Correlational matrix for the employees sample (*N* = 328).

**Variable**	**1**	**2**	**3**	**4**	**5**	**6**	**7**
1. Global self-esteem contingency	_						
2. Contingent upon competence	0.76	_					
3. Self-critical	0.80	0.49	_				
4. RBSE	0.73	0.34	0.31	_			
5. Self-esteem	−0.64	−0.40	−0.67	−0.37	_		
6. Self-oriented perfectionism	0.33	0.26	0.35	0.14	−0.12	_	
7. Need to belong	0.67	0.41	0.40	0.70	−0.44	0.24	_

All correlations are significant at *p* < 0.001 except correlation between self-oriented perfectionism and self-esteem (ns) and correlation between self-oriented and RBSE (*p* < 0.05). Only a subsample of 200 employees completed the self-esteem, self-oriented perfectionism, and need to belong scales.

#### 4.4.4 Temporal stability and predictive validity

To assess the temporal stability and predictive validity of the tool, we invited a subsample of our students sample 2 (*N* = 134, *N*women = 109, *N*men = 22, *N*other = 3; *M*age = 18.6; *SD* = 0.88) to complete the contingency scale and a stress scale for a second time, 6 weeks after the initial administration. The analysis of correlations between the two time points revealed a strong positive relationship between the measures of general self-esteem contingency (*r* = 0.86, *p* < 0.001), contingent upon competence (*r* = 0.73, *p* < 0.001), and Relation Based Self-Esteem (*r* = 0.78, *p* < 0.001), indicating good temporal stability of the scale. Regarding the predictive validity of the tool, we conducted regression analyses to predict the stress level at T2 from self-esteem contingency while controlling for the initial stress level of the students. Thus, the level of stress 6 weeks later was significantly predicted by contingency upon competence (β = 0.13, *p* < 0.05) and tendentially by general self-esteem contingency (β = 0.17, *p* = 0.06). In contrast, when controlling for the initial stress level, RBSE did not significantly predict stress at T2 (β = 0.10, *p* = 0.27). However, it is important to note that stress at T2 is primarily predicted by stress at T1 (β = 0.53, *p* = 0.001). This strong relationship suggests that the direct effect of self-esteem contingency on stress at 6 weeks is relatively limited. Additionally, stress at T1 is positively correlated with general contingency (*r* = 0.45, *p* < 0.001), contingency upon competence (*r* = 0.27, *p* < 0.01), and RBSE (*r* = 0.29, *p* < 0.001). Given the central role of stress at T1 in predicting stress at T2 and the correlations between self-esteem contingency and stress at T1, we hypothesized that the relationship between self-esteem contingency at T1 and stress at T2 might be mediated by stress at T1, which led us to explore potential mediating effects. The results of exploratory mediation analyses support this hypothesis and indicate that general self-esteem contingency (β = 0.22, *p* < 0.001) and contingency upon competence (β = 0.14, *p* < 0.01) both have a significant indirect effect on stress at T2, mediated by stress at T1. Furthermore, stress at T1 fully mediates the relationship between RBSE (β = 0.15, *p* < 0.01) and stress at T2, suggesting that the impact of Relation Based Self-Esteem on mid-term stress operates through short-term stress. In summary, the results show that self-esteem contingency has a moderate effect on students' stress at 6 weeks, with significant prediction of stress at T2 primarily through its indirect effect on short-term stress (T1). While the direct effect of self-esteem contingency on medium-term stress (T2) is limited, its influence seems to transfer through short-term stress (T1). Finally, RBSE seems to play a less central role in predicting mid-term stress than general or competence-related contingency, although its indirect influence through stress at T1 is notable.

## 5 General discussion

The three main objectives of this research were (1) to propose a simplified and shortened French version of the two self-esteem contingency scales by Johnson and Blom (2007), (2) to test its psychometric qualities and predictive validity, and (3) to extend its application to different populations (students, employees, and job seekers). The findings across studies validated the tool in these three groups, confirming its robust psychometric properties. In Study 1, an exploratory factor analysis conducted with students revealed two primary dimensions: Competence Based Self-Esteem (CBSE), and Relation Based Self-Esteem (RBSE). These findings aligned with Johnson and Blom's original conceptualization (Johnson and Blom, 2007) and formed the basis for the confirmatory factor analysis (CFA) in Study 2. The CFA demonstrated strong fit indices for the two-dimensional model, but a bifactorial model incorporating a global self-esteem contingency factor showed even better fit. A closer examination of the CBSE factor revealed opposing factor loadings between items reflecting “contingent upon competence” and “self-critical” subdimensions. Treating CBSE as a single factor failed to capture these nuances fully. By distinguishing these subdimensions, the three-factor bifactorial model—including “contingent upon competence,” “self-critical,” and RBSE—proved to be the best fit, with strong factor loadings and theoretical consistency. This result supports both a global approach, integrating self-criticism, and a more specific approach that separates competence-based and relationship-based contingencies. The scale also demonstrated good internal consistency, whether used globally or for individual factors, and showed temporal stability over 6 weeks. Moreover, this revised version provides clearer distinctions within self-esteem contingency. The global factor aligns with the conceptualization by Deci and Ryan (1995) and operationalized by Paradise and Kernis (1999), which emphasizes that self-esteem depends on meeting performance or acceptance criteria. This reliance can lead to vulnerability, as failure to meet these criteria may result in feelings of incompetence or shame, potentially undermining self-esteem. Notably, the self-critical dimension identified in the scale reflects this tendency, capturing how individuals internalize failure and engage in negative self-evaluation when these external standards are not met. Our revised version also retains (Johnson and Blom's, 2007) contributions by clearly differentiating contingencies based on competence and affiliation needs. In addition, correlations with other constructs confirmed the scale's convergent, discriminant, and concurrent validity. For example, the global measure of self-esteem contingency positively correlated with self-esteem instability and an extrinsically contingent orientation, while being negatively associated with overall self-esteem levels. Moreover, the contingency upon competence and RBSE factors showed distinct patterns of association with self-oriented perfectionism and the need for belonging, thereby supporting the discriminant validity of these dimensions. It is worth noting that the high correlation between RBSE and the need for belonging aligns with the sociometer theory (Leary and Baumeister, 2000), which posits that self-esteem serves as a gauge for social acceptance. According to this theory, lower social inclusion prompts efforts to restore social bonds, whereas higher self-esteem reduces concern for external approval. Thus, these findings underscore the importance of considering social context when studying self-esteem contingencies. However, despite the conceptual proximity between the scales of Sanquirgo et al. (2012) and those of Johnson and Blom (2007), these two measures differ in their purpose and operationalization. While the first measures the need for acceptance and closeness with others, the second focuses more on the link individuals establish between their self-esteem and the perceived acceptance or rejection.

Moreover, the predictive validity of the measure was confirmed through its relationship with stress levels over a 6-week period. Specifically, and in line with the findings of Crocker et al. (2003), contingency upon competence showed a stronger connection to stress compared to RBSE, highlighting the greater pressure linked to achievement-oriented contingencies. Although these findings are interesting, future research could benefit from testing the predictive validity of both types of contingencies on other variables, particularly regarding RBSE. While Johnson and Blom (2007) mention that compliance strategies are associated with this form of contingency, it would be worthwhile to further investigate this link. More precisely, it would be valuable to explore whether RBSE predicts the use of other reactions, such as anger and hostility, as highlighted in Kernis (2003) and Kernis and Lakey (2010)'s study on self-esteem contingency. On a different note, significant differences in self-esteem contingency were observed across different populations. For example, psychology students exhibited higher contingency upon competence than both employees and job seekers, likely due to the academic environment's emphasis on performance and approval. These findings suggest that students, who are often evaluated based on their competencies, may internalize the achievement standards set by teachers and/or parents, making their self-esteem more contingent upon their competencies (Deci and Ryan, 1995; Haines and Schutte, 2023). It is important to emphasize that this higher contingency, compared to the other two groups, is observed specifically among female students. From an early age, women are frequently socialized to excel in areas associated with caregiving and nurturing (Eagly and Koenig, 2021), which could lead them to place greater value on their skills in fields like psychology, which is often seen as a “feminine” domain. However, the noticeable gender imbalance in the number of participants represents a limitation of this study. A more balanced sample would offer a clearer and more accurate interpretation of the observed gender differences. Employees, who work in environments where their competencies are less directly evaluated, may be less inclined to internalize these external standards and may develop more intrinsic performance criteria (Hallsten et al., 2012). However, they are still likely to be concerned with gaining others' approval, as work is a central socialization domain (Deci and Ryan, 2008). Lastly, it is surprising to find that job seekers exhibit less contingent self-esteem than both students and employees. This contradicts the usual literature, which emphasizes the negative impact of unemployment on psychological health, including self-esteem (Paul and Moser, 2009), often due to rejection and discrimination (Bourguignon and Herman, 2007). However, it is important to note that, like other members of stigmatized groups, job seekers typically do not remain passive when their identity is threatened and implement strategies to protect themselves. These strategies, such as minimizing discrimination or engaging in psychological disengagement, may help explain why job seekers appear less vulnerable to rejection (Bourguignon and Herman, 2006; Major et al., 1998). The validation of this contingency scale opens up interesting opportunities for future research and practical applications. Grounded in self-determination theory (Ryan and Deci, 2017), it provides valuable insights into the antecedents of self-esteem contingencies. While frustrating environments and conditional parental love are often cited as contributing factors (Deci and Ryan, 1995; Haines and Schutte, 2023), further exploration of how contexts that frustrate the need for competence and/or affiliation foster self-esteem contingency related to those needs would be particularly valuable. Testing the direct link between the frustration or satisfaction of fundamental needs and self-esteem contingency, as well as exploring the impact of this contingency on individuals' motivations, offers a promising avenue for future research. Additionally, examining how the contextual satisfaction or frustration of fundamental needs influences this contingency could provide practical insights for educators, managers, and employment counselors.

## Data Availability

The original contributions presented in the study are included in the article/Supplementary material, further inquiries can be directed to the corresponding author.
